# Supplementary material for: Clinical Snapshot of Group A Streptococcal Isolates from an Australian Tertiary Hospital
Source: Pathogens. 2024 Nov 1;13(11):956. doi: 10.3390/pathogens13110956 (PMC11597359; doi:10.3390/pathogens13110956)
Supplement: Supplementary file 1 [file pathogens-13-00956-s001.zip › pathogens-3275597-supplementary.pdf]

**Supplementary Material**

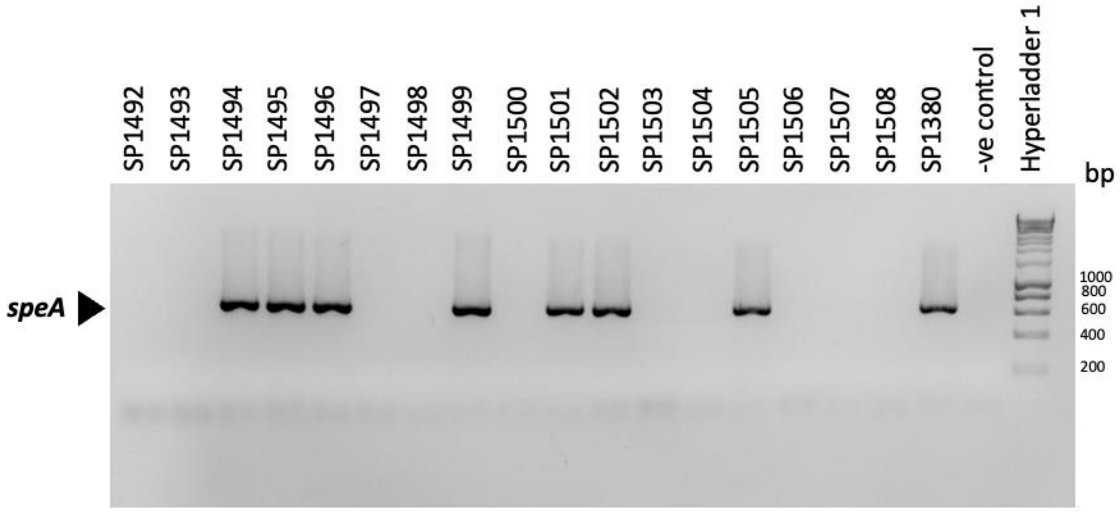


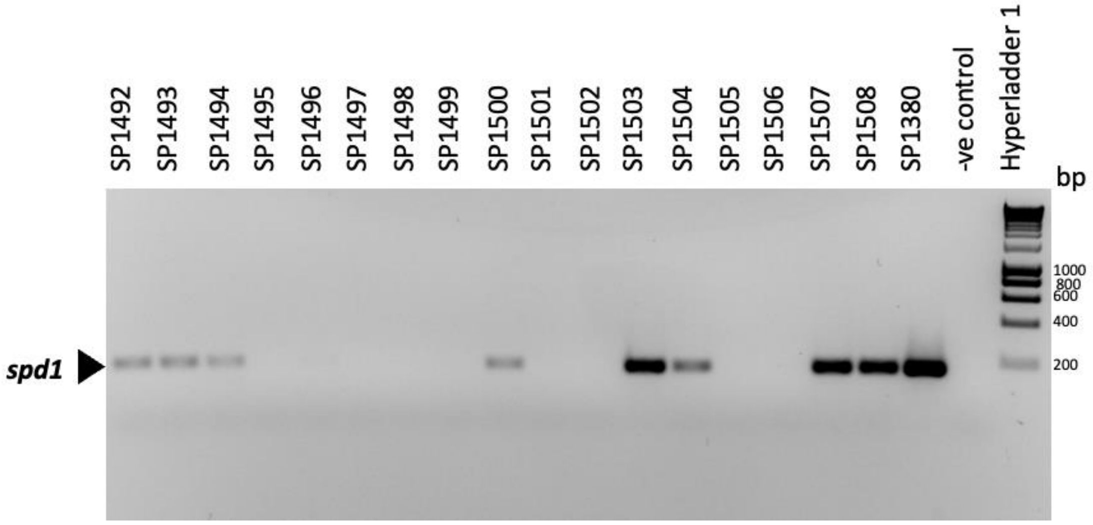


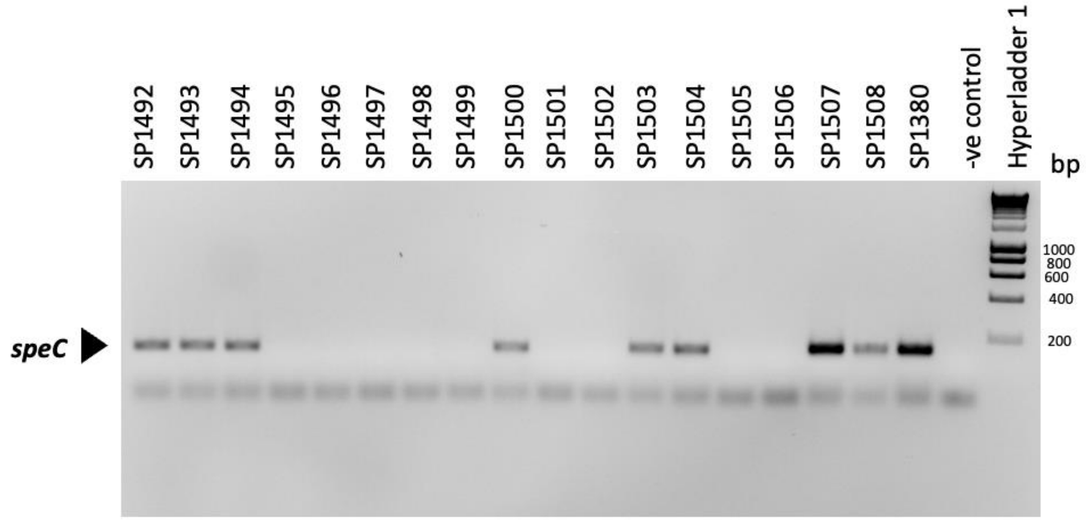

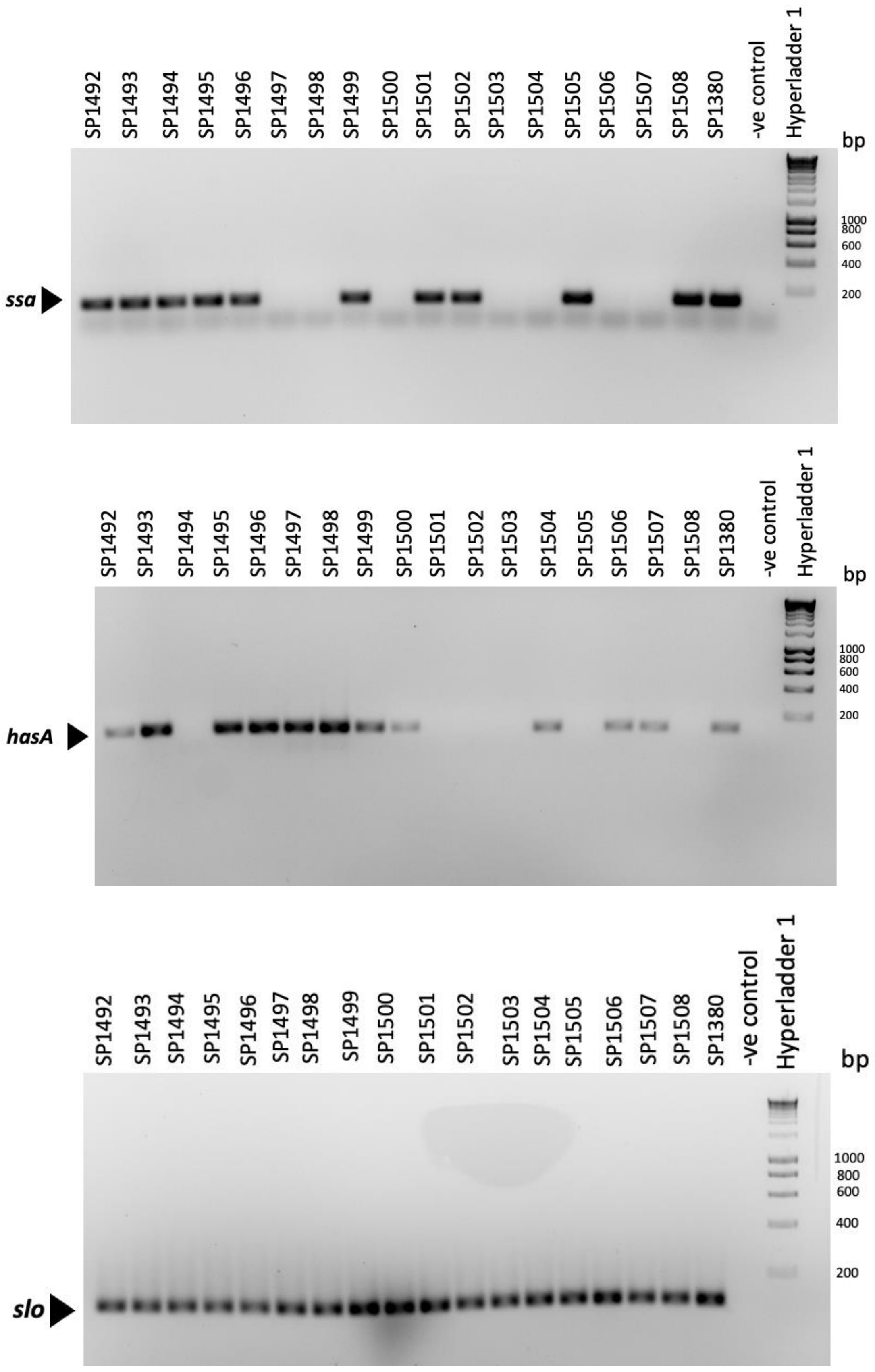

SP1492
SP1493
SP1494
SP1495
SP1496
SP1497
SP1498
SP1499
SP1500
SP1501
SP1502
SP1503
SP1504
SP1505
SP1506
SP1507
SP1508
SP1380
-ve control
Hyperladder 1
bp
1000
800
600
400
200
ssa
hasA
slo

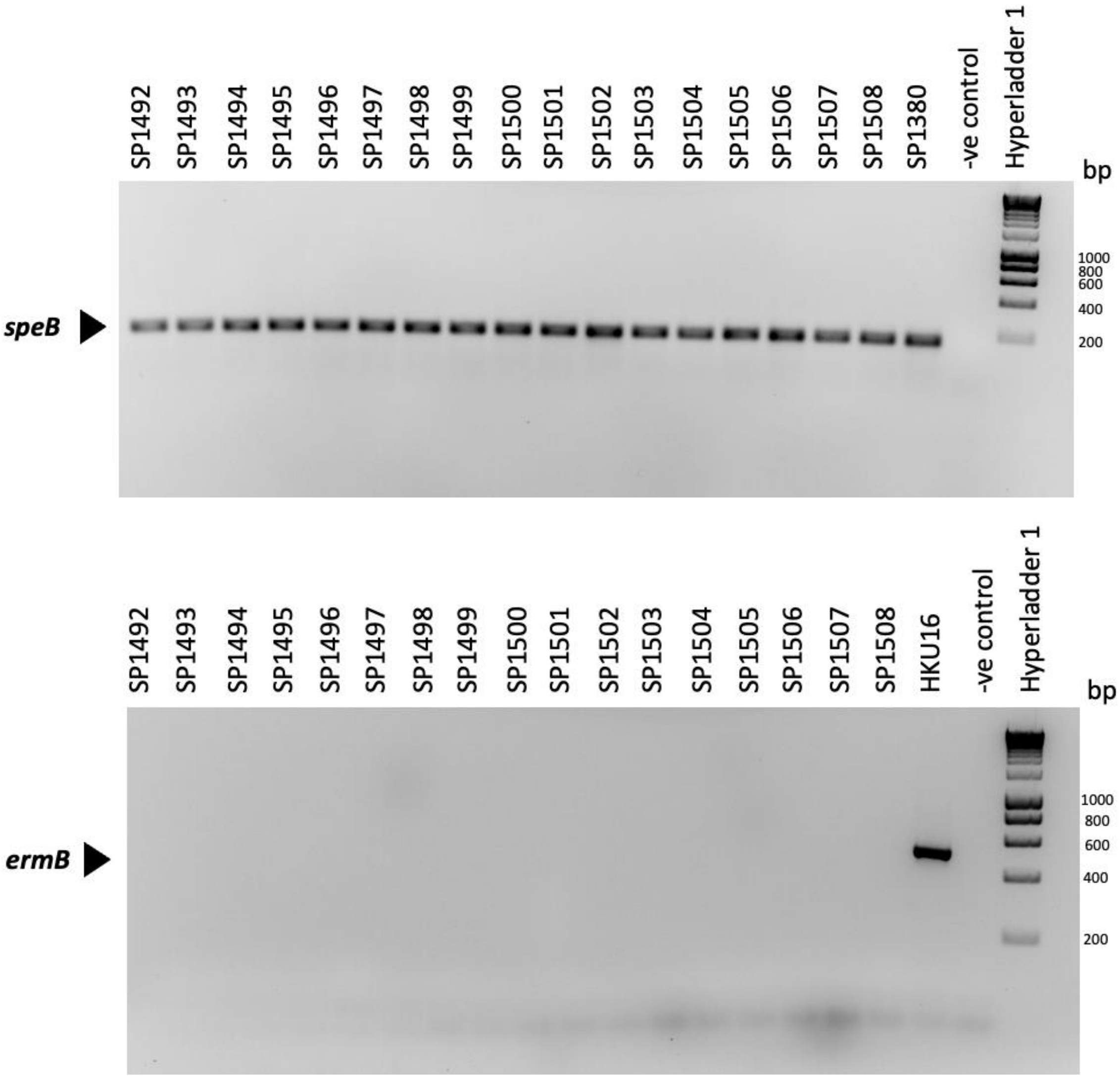


**Supplementary Figure S1.** Images of agarose gels following polymerase chain reaction amplification of toxin and antibiotic resistance genes from 17 clinical Group A *Streptococcus* isolates from Gold Coast University Hospital. SP1380 and HKU16 served as positive controls and distilled $H_2O$ was used as a negative control. HyperLadder™ 1kb (Meridian Bioscience #BIO-33053) was used as molecular weight marker.

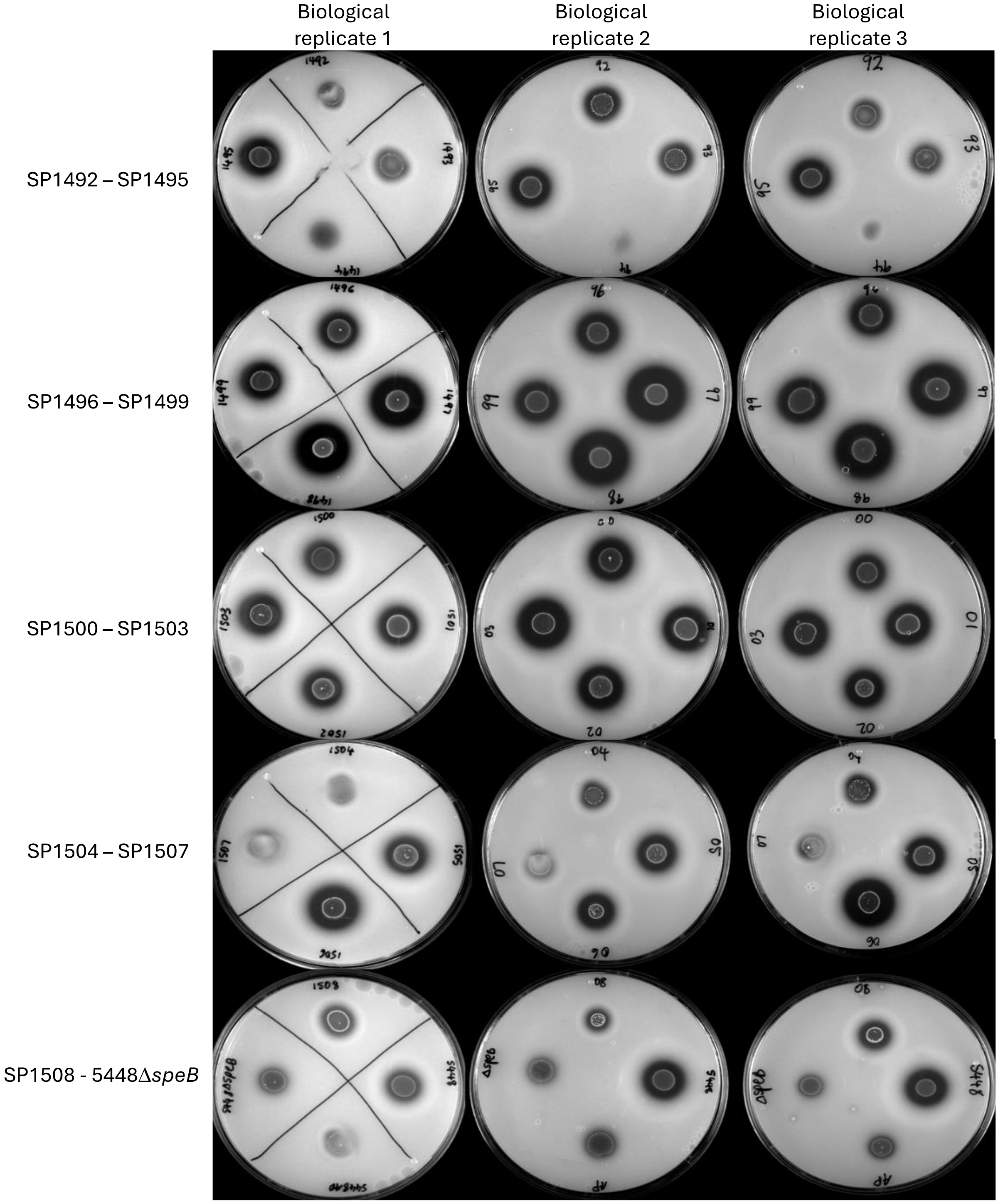


**Supplementary Figure S2.** Results from the streptococcal pyrogenic exotoxin B (SpeB) caseinolytic activity assay for 17 clinical isolates from Gold Coast University Hospital. SpeB activity is indicated by a clear zone with an opaque halo surrounding the culture on the plate. Positive control: 5448; negative controls: 5448Δ*speB* and 5448AP.

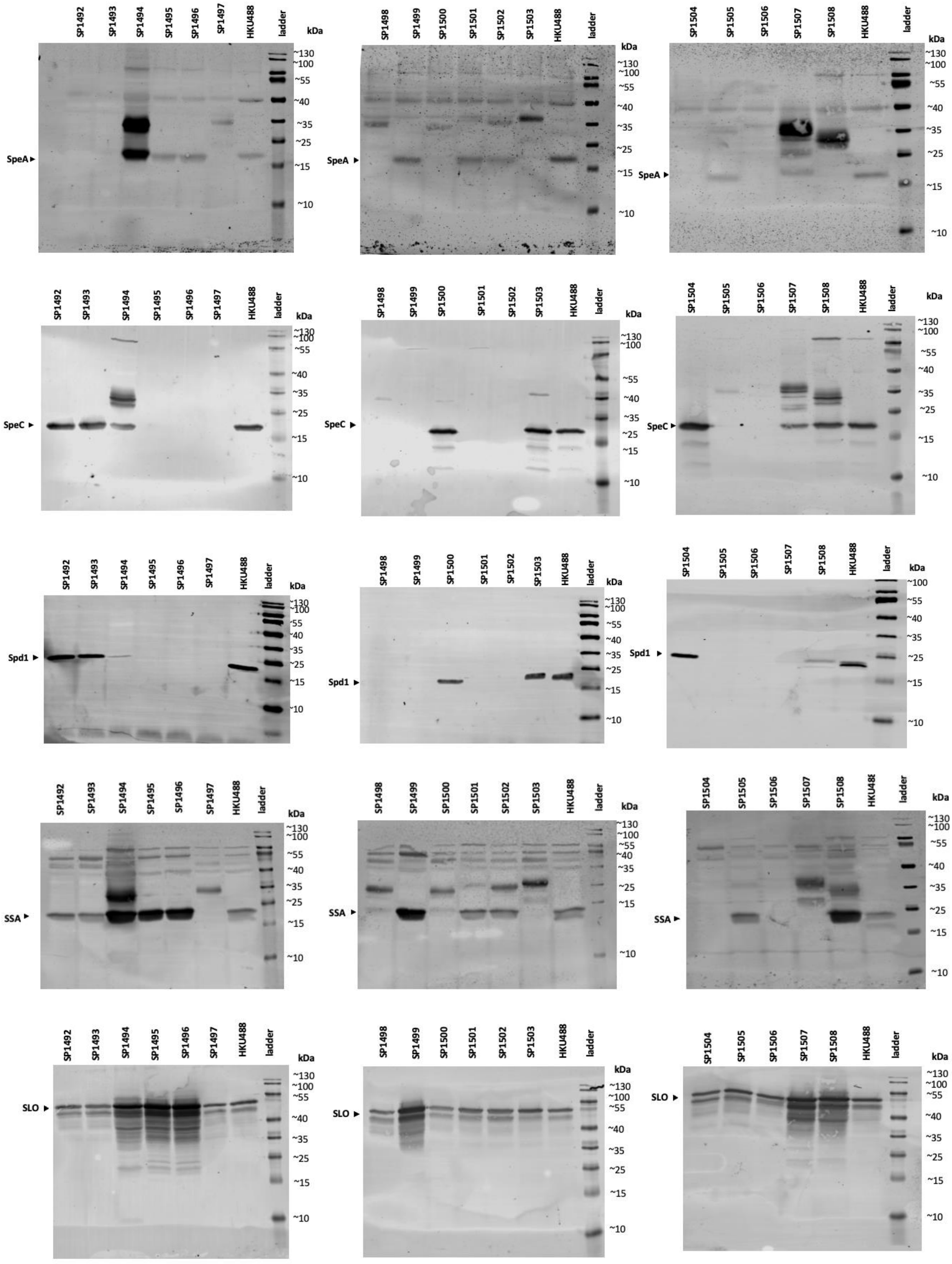
SP1492 SP1493 SP1494 SP1495 SP1496 SP1497 HKU488 ladder
SP1498 SP1499 SP1500 SP1501 SP1502 SP1503 HKU488 ladder
SP1504 SP1505 SP1506 SP1507 SP1508 HKU488 ladder
kDa
~130 ~100 ~55 ~40 ~35 ~25 ~15 ~10
SpeA
SpeC
Spd1
SSA
SLO

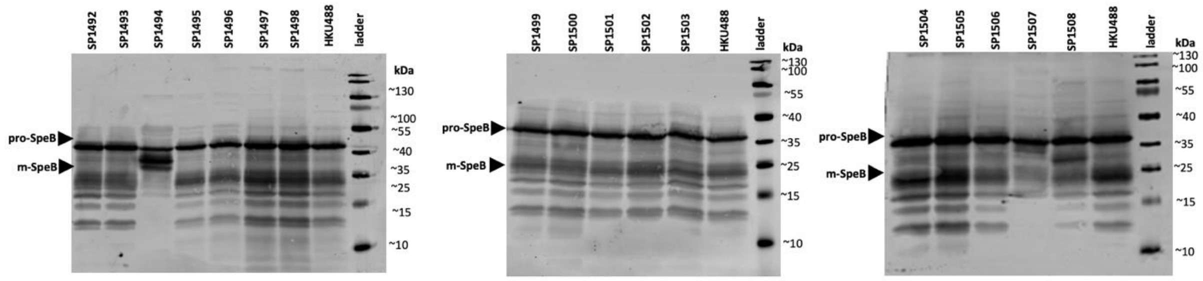


**Supplementary Figure S3**. Images of Western blots for the detection of Group A *Streptococcus* toxins in supernatant from the 17 clinical isolates from the Gold Coast University Hospital. Isolate HKU488 was used as positive control. PageRuler™ pre-stained protein ladder (ThermoScientific #26616) was used as molecular weight marker.

**Supplementary Table S1.** Primers for polymerase chain reaction screening of Group A streptococcal scarlet fever toxins and antibiotic resistance genes.

| Primer ID | Primer sequence (5′ - 3′ orientation) | Reference |
|---|---|---|
| speA-fw | GTGACATTTCTTGGACTAACAATCTCG | [1] |
| speA-rv | TATCATAAGATATTTAGATTGAGTAAATTCTGGTTCAG | |
| spd1-fw | CGTGGGCATCTAGTCGGATA | [2] |
| spd1-rv | GGTGAAGTGCAAGCCAAGAA | |
| speC-fw | GAATGTTAAAAGTGATTTACTTTATGCATACACTATAAC TC | [1] |
| speC-rv | CTCATAAGACATTTCGGAACTAATATAATAGTC | |
| ssa-fw | CAGAACAATTAAACAAATCTAGCCAATTTACTG | [1] |
| ssa-rv | GAAAAATCAAATCATGCTGTAAAAGCTGAC | |
| hasA-fw | GGGAGTTCAAACACAGATGCA | [2] |
| hasA-rv | CTTGAGCATGGCGTTTTCCT | |
| SLO-fw | GCTGGCTAATAAAGGTTTTACCG | [3] |
| SLO_rv | CGGTAAAACCTTTATTAGCCAGC | |
| speB-fw | TGCTGACGGACGTAACTTCT | [3] |
| speB-rv | CCACCAGTACCAAGAGCTGA | |
| ermB-fw | CATTGCTTGATGAAACTGATTTTTAGTAAACAG | [1] |
| ermB-rv | CGAAACTGGCTAAAATAAGTAAACAGG | |

**Supplementary Table S2.** Summary of SpeB caseinolytic assay results.

| Isolate | SpeB activity |
|---|---|
| SP1492 | + |
| SP1493 | + |
| SP1494 | Ind[a] |
| SP1495 | + |
| SP1496 | + |
| SP1497 | + |
| SP1498 | + |
| SP1499 | + |
| SP1500 | + |
| SP1501 | + |
| SP1502 | + |
| SP1503 | + |
| SP1504 | + |
| SP1505 | + |
| SP1506 | + |
| SP1507 | + |
| SP1508 | + |
| 5448 | + |
| 5448AP | - |
| 5448Δ*speB* | - |

SpeB, streptococcal pyrogenic exotoxin B; +, positive; -, negative; Ind, indefinite.
[a] Isolate displayed inconsistent results across three biological replicates. Inconsistency is likely due to the growth defect and m-SpeB phenotype associated with the isolate.

## References

1. Walker, M.J.; Brouwer, S.; Forde, B.M.; Worthing, K.A.; McIntyre, L.; Sundac, L.; Maloney, S.; Roberts, L.W.; Barnett, T.C.; Richter, J.; et al. Detection of epidemic scarlet fever Group A *Streptococcus* in Australia. *Clin. Infect. Dis.* **2019**, *69*, 1232-1234.
2. Brouwer, S.; Das, S.; Hayes, A.J.; Bertolla, O.M.; Davies, M.R.; Walker, M.J.; Whiley, D.M.; Irwin, A.D.; Tickner, J.A. A rapid molecular detection tool for toxigenic M1UK *Streptococcus pyogenes*. *J. Infect. Dis.* **2024**.
3. Brouwer S, Brouwer, S.; Jespersen, M.G.; Ong, C.Y.; De Oliveira, D.M.P.; Keller, B.; Cork, A.J.; Djoko, K.Y.; Davies, M.R.; Walker, M.J. *Streptococcus pyogenes* Hijacks Host Glutathione for Growth and Innate Immune Evasion. *mBio* **2022**, *13*, e0067622.

**Disclaimer/Publisher's Note:** The statements, opinions and data contained in all publications are solely those of the individual author(s) and contributor(s) and not of MDPI and/or the editor(s). MDPI and/or the editor(s) disclaim responsibility for any injury to people or property resulting from any ideas, methods, instructions or products referred to in the content.
